# Construção e Validação do Protocolo EmpoderACO Direcionado a Pacientes em Anticoagulação Oral com Varfarina

**DOI:** 10.36660/abc.20220576

**Published:** 2023-06-20

**Authors:** Hannah Cardoso Barbosa, Heloisa de Carvalho Torres, João Antônio de Queiroz Oliveira, Rebeca Priscila de Melo Santos, Josiane Moreira da Costa, Leonardo Gonçalves Miranda, Adriana Silvina Pagano, Marcus Fernando da Silva Praxedes, Maria Auxiliadora Parreiras Martins

**Affiliations:** 1 Universidade Federal de Minas Gerais Belo Horizonte MG Brasil Universidade Federal de Minas Gerais, Belo Horizonte, MG – Brasil; 2 Hospital Risoleta Tolentino Neves Belo Horizonte MG Brasil Hospital Risoleta Tolentino Neves, Belo Horizonte, MG – Brasil; 3 Universidade Federal dos Vales do Jequitinhonha e Mucuri Teófilo Otoni MG Brasil Universidade Federal dos Vales do Jequitinhonha e Mucuri, Teófilo Otoni, MG – Brasil; 4 Universidade Federal do Recôncavo da Bahia Cruz das Alma BA Brasil Universidade Federal do Recôncavo da Bahia (UFRB), Cruz das Almas, BA – Brasil

**Keywords:** Comportamentos Relacionados com a Saúde, Educação em Saúde, Varfarina, Anticoagulante

## Abstract

**Fundamento:**

A varfarina é um anticoagulante oral útil para prevenção de tromboembolismo, embora seja considerado fármaco de alto risco de causar eventos adversos. Considerando os desafios práticos no controle da anticoagulação oral, os pacientes poderiam se beneficiar de estratégias educacionais que visem mudança de comportamento, participação ativa no autocuidado e adesão à farmacoterapia.

**Objetivo:**

Construir e validar o protocolo EmpoderACO para mudança de comportamento em pacientes em uso de varfarina.

**Métodos:**

As etapas metodológicas foram: definição de conceitos e domínios do autocuidado, identificação dos objetivos, construção e seleção dos itens, avaliação da validade de conteúdo e pré-teste na população alvo.

**Resultados:**

Relevância, adequação, clareza e confiabilidade interna dos itens do instrumento foram avaliadas por comitê de juízes multiprofissional pela plataforma web E-surv, obtendo-se média de concordância ≥0,91. A compreensão do instrumento pela população-alvo teve clareza adequada com média de 0,96.

**Conclusão:**

O EmpoderACO poderá contribuir para qualificar o processo de comunicação entre profissionais e pacientes, melhorar a adesão ao tratamento e os resultados clínicos, podendo ser replicado nos serviços de saúde.

## Introdução

A varfarina é um anticoagulante oral derivado cumarínico de uso amplo no mundo para prevenção primária e secundária de tromboembolismo.^1^ Mesmo com o advento dos anticoagulantes orais diretos, a varfarina ainda é o principal anticoagulante oral distribuído pelo Sistema Único de Saúde do Brasil.^2^ Entretanto, o controle inadequado do uso desse medicamento pode propiciar a ocorrência de eventos adversos, representados por hemorragia e tromboembolismo que podem ocorrer devido à exacerbação do efeito anticoagulante ou falência terapêutica, respectivamente.^3,4^ O emprego de intervenções educacionais pode contribuir para o alcance dos objetivos terapêuticos do uso desse medicamento, favorecendo o aumento do conhecimento do paciente sobre anticoagulação oral, com consequente melhoria da adesão e satisfação com seu tratamento.^5-8^ Mudança de comportamento desejável, ações direcionadas ao letramento em saúde e empoderamento dos pacientes têm sido apontados como elementos importantes para o sucesso do tratamento anticoagulante.^9-11^

O tratamento requer monitorização laboratorial frequente e o alcance da faixa terapêutica alvo pode ser difícil, devido aos múltiplos fatores interferentes do tratamento, tais como: grande variabilidade dose-resposta, influência de polimorfismo genético, presença de comorbidades, elevado número de interações com medicamentos e alimentos, baixa adesão ao tratamento, baixo letramento em saúde e receio das reações adversas por parte dos pacientes que podem levar à não administração ou interrupção do medicamento por conta própria, podendo exigir ajustes posológicos frequentes.^1,7,12-14^ O risco de ocorrência de eventos adversos ao tratamento torna-se maior quando há o uso incorreto deste medicamento, os quais podem ser representados por eventos hemorrágicos graves como o acidente vascular cerebral hemorrágico, e/ou trombóticos, sendo estes eventos desencadeados pela exacerbação do efeito anticoagulante ou falência terapêutica, respectivamente.^1,7,11,15,16^Nesse contexto, a adesão à farmacoterapia se estabelece, ainda, como condição necessária para melhorar a efetividade e segurança do tratamento, trazendo desafios adicionais para o processo de cuidado ao paciente.^16^

O empoderamento mostra-se útil para o aumento da sensação de controle, autoeficácia, habilidades de enfrentamento, gerenciamento do tratamento e capacidade do indivíduo de refletir sobre sua contribuição no processo de cuidado, além de alcançar uma mudança de comportamento sobre sua própria condição de saúde.^17-20^ No contexto de doença crônicas, foi proposto o Protocolo Mudança de Comportamento (PMC) originalmente desenvolvido por pesquisadores da Universidade de Michigan para pacientes com diabetes *mellitus* tipo 2,^21-23^ e posteriormente traduzido e validado para a população brasileira.^24,25^ Atualmente, há carência de instrumentos e diretrizes que guiem as práticas com abordagem no empoderamento, autocuidado e mudança de comportamento dos pacientes na área da anticoagulação, além dos profissionais da saúde nem sempre estarem conscientizados e mobilizados sobre a importância desse tipo de abordagem educacional.^9,25^

Neste sentido, a utilização de estratégias padronizadas pautadas no empoderamento poderá nortear os profissionais de saúde no estímulo da participação ativa dos pacientes no autocuidado e na adesão à farmacoterapia dos pacientes com cardiopatias em anticoagulação oral. A construção de um protocolo voltado para a anticoagulação oral com varfarina, baseado nos princípios de mudança de comportamento do PMC, poderia promover melhores resultados clínicos e uma sistematização do canal de comunicação entre o paciente e o profissional de saúde. Além disso, poderia aumentar a satisfação do paciente com seu tratamento, melhorar a adesão, reduzir a ocorrência de eventos adversos e possibilitar que o usuário/paciente reconheça a necessidade da mudança de comportamento.^7,26,28^ O objetivo deste trabalho foi construir e validar o protocolo EmpoderACO voltado para a mudança de comportamento de pacientes em anticoagulação oral com varfarina.

## Métodos

O estudo foi desenvolvido em etapas seguindo a metodologia proposta por Coluci et al.,^26^ a saber: definição de conceitos e domínios do autocuidado na anticoagulação com varfarina; identificação dos objetivos do instrumento; construção do instrumento e seleção dos itens conforme o objetivo do instrumento; avaliação da validade de conteúdo pelo Comitê de Juízes (CJ); realização da validação pré-teste em pacientes em uso de varfarina e descrição das variáveis e análise estatística. As etapas do estudo ocorreram no período de dezembro de 2017 a junho de 2019.

A estrutura dos itens do protocolo EmpoderACO seguiram os cinco passos para mudança de comportamento, conforme o estudo de empoderamento PMC,^21-25^ a saber: 1º Passo: definição do problema; 2º Passo: identificação e abordagem dos sentimentos; 3º Passo: definição de metas; 4º Passo: elaboração do plano de cuidados para conquista da(s) meta(s); 5º Passo: Avaliação e experiência do usuário sobre o plano de cuidados. A pesquisa foi aprovada pelo Comitê de Ética em Pesquisa da Universidade Federal de Minas Gerais (UFMG), sob parecer nº 2.018.850, CAAE: 65928316.3.0000.5149. Após esclarecimentos sobre o objetivo da investigação e a natureza da coleta de dados, todos os participantes assinaram o termo de consentimento livre e esclarecido.

### Definição de conceitos, domínios do autocuidado e objetivos do instrumento

De acordo com o Pasquali,^29^ a construção de uma estrutura conceitual é a etapa responsável por definir o contexto do instrumento e sustentar o desenvolvimento de sua dimensionalidade. Desta forma, um mapa conceitual foi construído, utilizando o programa *CmapTolls* versão 6.02 (2017), para a identificação dos domínios do autocuidado nos quais o instrumento deveria se embasar. Identificou-se a necessidade de construção de itens específicos para o público-alvo envolvendo pacientes com cardiopatias com alta complexidade do quadro clínico e especificidades inerente ao anticoagulante oral. Estas etapas foram conduzidas no período de dezembro de 2017 a agosto de 2018. De acordo com o estudo de Snyder et al.,^30^ é fundamental que os objetivos do instrumento de saúde sejam pré-definidos antes de sua construção e que esses objetivos tenham conexão com domínios e conceitos a serem inseridos no instrumento.

### Construção do instrumento e seleção dos itens

A construção e validação do instrumento, em questão, foram realizadas seguindo etapas metodológicas abordadas propostas pelos estudos de Coluci et al.^26^ e Pasquali.^29^ Incialmente, o instrumento foi construído por um comitê de especialistas (CE) interno, o qual apresentava ampla vivência em clínica de anticoagulação, composto por três farmacêuticas clínicas, uma enfermeira e uma linguista com domínio no processo de adaptação e validação de instrumentos empregados na área da saúde. Foram conduzidas reuniões para discussão da pertinência e adequação de cada item para o contexto da anticoagulação oral. Coube ao CE interno a definição dos domínios de autocuidados, mensurados na etapa anterior do mapa conceitual, a serem incluídos no protocolo. Observou-se a necessidade da elaboração pelo CE interno, de 12 novos itens para que o novo instrumento atendesse à população-alvo e estivesse voltado para o autocuidado na anticoagulação oral. Nesta fase, foram construídas oito versões diferentes do protocolo (V1-V8) antes do encaminhamento ao CE externo (Material Suplementar A).

A versão V8 definida como versão-teste foi submetida à avaliação piloto do CE externo por meio da plataforma web *Survey E-surv*. Nesta avaliação piloto, foram convidados cinco profissionais da área da saúde, todos com conhecimento e experiência em anticoagulação composto por um médico, dois farmacêuticos e dois enfermeiros. O CE externo analisou cada item do protocolo e sugeriu novas adequações na estrutura e conteúdo. Após as adaptações sugeridas e julgadas pertinentes pelo CE interno, foi construída uma versão inicial do instrumento (V9) que, posteriormente, foi entregue a ao Comitê de Juízes (CJ). Estas etapas foram conduzidas no período de setembro de 2018 a março de 2019.

### Avaliação do instrumento por CJ

O CJ estava composto por 34 profissionais e tinha um perfil multidisciplinar, cuja função era o julgamento e a análise de todos os itens do protocolo. Posteriormente, a estruturação e organização do instrumento foram testadas quanto à hipótese de que os itens escolhidos contemplavam adequadamente os domínios do constructo desejado.^26,29,31^ O CJ realizou esta análise por meio da avaliação de conteúdo, seguindo a recomendação da literatura em relação ao número mínimo de juízes e composição de especialistas na área de instrumentos de medidas.^32^ As análises pelos juízes envolveram procedimentos avaliativos e quantitativos.^26^ A seleção dos profissionais que integraram o CJ foi realizada considerando os seguintes critérios: possuir graduação na área da Saúde e apresentar conhecimento e/ou vivência com a prática clínica envolvendo cuidado ao paciente em anticoagulação oral com varfarina e/ou profissionais com experiência no processo de adaptação e validação de instrumentos.

Os integrantes do CJ preencheram um questionário introdutório por meio da mesma plataforma *online* de avaliação (*Survey E-surv)*. Em seguida, os participantes foram convidados a avaliar a versão V9 e registrar suas opiniões a fim de avaliar o grau de relevância, adequação e clareza do instrumento, tendo estipulado prazo de um mês para o retorno das avaliações. Também foi solicitado ao CJ que analisasse o grau de pertinência dos itens do protocolo e informasse qual(is) categoria(s) o item era capaz de mensurar. Nessa análise de pertinência, o juiz tinha acesso ao significado de cada categoria e poderia selecionar mais de uma categoria correspondente para o mesmo item. O objetivo dessa análise foi agrupar os itens de acordo com os domínios de autocuidado para pacientes em uso de anticoagulante oral. Estas etapas foram conduzidas no período entre março e abril de 2019.

### Validação pré-teste em pacientes

A análise semântica e validação do instrumento foram realizadas por meio do pré-teste que consistiu em um teste de campo com a população-alvo utilizando a versão pré-final do instrumento (V10). Os participantes avaliaram a clareza de cada item do instrumento a fim de estimar o entendimento do instrumento. Esta etapa foi realizada na clínica de anticoagulação do Hospital das Clínicas - UFMG por dois pesquisadores da área da saúde experientes em aplicação de questionário para pacientes. O pré-teste foi aplicado em 30 pacientes. As questões foram lidas aos participantes pelos pesquisadores, pois alguns deles não tinham letramento suficiente. Coube aos participantes responderem quanto à clareza dos itens, em uma escala tipo *Likert* de três pontos: a) Muito claro, b) Claro e c) Pouco claro. Estas etapas foram conduzidas no período entre abril e maio de 2019. Após o pré-teste não houve necessidade de modificação dos itens construídos e adaptados e, portanto, a versão V10 constituiu a versão final do protocolo EmpoderACO.

### Descrição das variáveis

Os dados descritivos dos integrantes do CJ foram coletados por meio da aplicação de questionário inicial, via plataforma web *Survey E-surv*. Esses dados incluíram: nome, instituição de trabalho, formação e experiência na prática profissional com varfarina. As avaliações do instrumento obtidas pelo CJ foram exportadas da plataforma *online* para uma planilha eletrônica do editor Microsoft Excel^R^ (versão 2019) para posterior realização das análises estatísticas. Todos os dados foram devidamente codificados para assegurar o anonimato dos participantes.

Durante o pré-teste, foi aplicado um questionário para coleta dos dados sociodemográficos dos pacientes, abrangendo sexo, idade e escolaridade, de forma de caracterizar a amostra. Esta etapa foi conduzida em junho de 2019.

### Análise estatística

A validação do instrumento, neste estudo, foi computada por meio do Coeficiente de validade de conteúdo (CVC) por ser uma medida capaz de avaliar a relevância e representatividade dos itens. Foi definida uma concordância mínima de CVC igual a 0,80^33^ e, preferencialmente superior a 0,90.^34^ Para avaliar a relevância de cada item do instrumento, o CJ julgou o item de acordo com as respostas: 1=Sem relevância, 2=Relevante, 3=Muito relevante. A adequação e clareza foram avaliadas seguindo uma escala *Likert* de três pontos, sendo: 1=Não adequado, 2=Adequado e 3=Muito adequado, para o grau de adequação e 1=Sem clareza, 2=Claro e 3=Muito claro, para o grau de clareza. O cálculo foi realizado a partir do somatório das respostas “2” e “3” de cada juiz em cada item do protocolo e dividiu-se esta soma pelo número total de juízes (adaptado do estudo de Coluci et al.^26^). A etapa do pré-teste foi realizada por 30 pacientes, cujo quantitativo amostral foi considerado suficiente para tal avaliação.^29^ O mesmo cálculo de CVC foi utilizado na fase pré-teste aplicada em campo para a avaliação quanto à clareza dos itens. Os dados sociodemográficos foram tabulados e apresentados de modo descritivo utilizando frequências absoluta e relativa com cálculo de proporções e medidas de tendência central. Esta etapa foi conduzida em junho de 2019.

## Resultados

**Figura 1:**
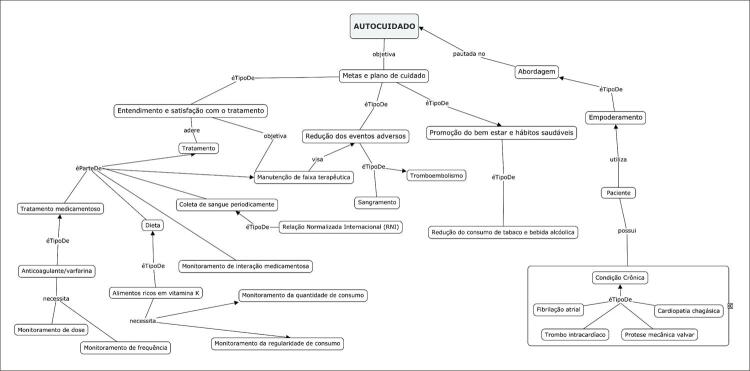
– Domínios do autocuidado em anticoagulação oral. Fonte: Elaborado com auxílio do CmapTolls.

Os domínios do autocuidado representados no mapa conceitual (Figura 1) foram divididos pelo CE interno em três categorias: 1) entendimento e satisfação com o tratamento; 2) redução dos eventos adversos e 3) promoção do bem-estar e hábitos saudáveis. Pela análise dos domínios e das categorias, observou-se a necessidade da construção de 12 novos itens para o protocolo, sendo eles: 4-10; 13-17, conforme apresentado na Tabela 1.

**Tabela 1:** – Coeficiente de validade de conteúdo das respostas do comitê de juízes da versão V8

Itens originais em português	Grau de Relevância (CVC)	Grau de Adequação (CVC)	Grau de Clareza (CVC)
1 – Qual é a sua maior dificuldade no controle do anticoagulante?	1,00	1,00	0,94
2 – Você poderia explicar essa dificuldade?	1,00	1,00	0,94
3 – Conte alguma situação que aconteceu com você por causa dessa dificuldade.	0,97	0,97	1,00
4 – Você toma a varfarina da forma como te orientaram?*	0,91	0,88	0,67**
5 – Você já interrompeu alguma vez seu tratamento com a varfarina? Por que?*	0,91	0,94	0,88
6 – Você acha que a alimentação pode atrapalhar o tratamento com varfarina?*	0,97	0,97	1,00
7 – Com que frequência e quantidade você consome verdura e folhas verdes?*	0,94	0,88	0,97
8 – Você faz uso de cigarro ou bebida alcoólica? Qual quantidade e frequência?*	0,91	0,82	0,94
9 – Você acha que usar outros medicamentos pode atrapalhar seu controle da anticoagulação?*	0,97	0,97	0,91
10 – Quando você identifica um sangramento o que você faz?*	1,00	1,00	0,97
11 – O que você acha de ter que tomar anticoagulante?	0,97	0,97	0,94
12 – Você acha que usar a varfarina pode fazer mal?*	0,85	0,91	0,97
13 – Você deixou de fazer coisas que você gostava de fazer depois que começou a tomar varfarina?	0,85	0,85	0,91
14 – O que mais incomodou você após o início da terapia com varfarina?	0,88	0,85	0,88
15 – Como você se sente tendo que fazer coletas de sangue frequentes?*	0,97	0,94	1,00
16 – Você acredita que meditação ou oração pode melhorar seu tratamento?*	0,76**	0,70**	0,82
17 – Quais são seus objetivos com o tratamento varfarina?*	0,94	0,91	0,90
18 – O que você acha que pode fazer para melhorar o seu tratamento?	0,97	1,00	0,97
19 – Como você pode mudar alguma coisa na sua vida para se sentir melhor?	0,70**	0,64**	0,67**
20 – O que você acha que pode atrapalhar para conseguir alcançar seus objetivos no tratamento?	0,91	0,94	0,91
21 – Tem alguma pessoa que pode ajudar no seu tratamento?	0,91	0,91	0,91
22 – Você sabe o que pode ter se não se cuidar?	0,97	0,85	0,73**
23 – Vamos juntos montar um plano para cuidar da sua saúde?	0,94	0,97	0,88
24 – Fale sobre o passo-a-passo que você pode fazer para melhorar o seu tratamento.	0,76**	0,79	0,88
25 – E o que você realmente vai fazer para melhorar?	0,88	0,88	0,88
26 – Quando você vai começar?	0,85	0,88	0,88
27 – O que você aprendeu com essa experiência?	0,91	0,94	0,82
28 – Que dificuldades você teve para seguir o plano?	0,91	0,91	0,85
29 – O que você faria de diferente da próxima vez?	0,82	0,85	0,82
30 – Você terminou o plano, e agora, o que você vai fazer?	0,79	0,79	0,82
**Média de CVC**	**0,92**	**0,91**	**0,91**

CVC: coeficiente de validade de conteúdo. * Itens totalmente construídos. ** Itens com CVC ≤0,78.

Dos 80 profissionais convidados para participar do CJ, 34 (42,5%) enviaram as avaliações do protocolo e o número total de juízes participantes mostrou ser adequado, conforme preconizado pela literatura.^35^ A distribuição das categorias profissionais dos participantes do estudo foi: 40 (50,0%) farmacêuticos; 17 (21,3%) enfermeiros; 16 (20,0%) médicos; três (3,7%) nutricionistas; três (3,7%) linguistas e uma pedagoga (1,3%). Os farmacêuticos representaram a categoria profissional de maior predominância no CJ, seguidos de enfermeiros, médicos, nutricionistas, linguistas e pedagoga. A média geral dos itens avaliados pelo CJ apresentou CVC igual ou superior a 0,91 para todas as análises: grau de relevância, adequação e clareza. As exceções foram quanto aos itens 4, 16, 19, 22 e 24, conforme apresentado na Tabela 1. Três itens da versão V9 foram excluídos após análise do CJ, análise qualitativa e quantitativa do CE interno, sendo eles 19, 24 e 25 por não apresentarem novos ganhos ao instrumento.

Verificou-se que houve consistência e homogeneidade na análise do grau de pertinência da versão V9, feita pelo CJ. Os resultados da concordância estão apresentados na Tabela 2.

**Tabela 2:** – Representação da concordância dos itens por categoria

Categoria	Itens
Adesão ao esquema posológico da terapia anticoagulante	1-5; 11-13; 15-21; 23-30
Coleta de sangue para o exame de monitorização da anticoagulação oral (RNI)	14
Consumo de alimentos que interferem no efeito anticoagulante	1-3; 6-7
Receio com a ocorrência de eventos adversos ao tratamento	3; 5; 8; 10; 12; 15; 22
Interações medicamentosas com a varfarina	9
Efetividade da farmacoterapia	17-18; 20; 22-26; 29-30
“Outros” (Entendimento e satisfação do tratamento)	19

RNI: Relação Normalizada Internacional.

A etapa do pré-teste foi realizada com 30 pacientes que representaram uma amostra heterogênea quanto a idade, sexo e nível de escolaridade, sendo que, 50,0% dos participantes corresponderam ao sexo feminino e 50,0% tinham escolaridade no nível do ensino fundamental incompleto. A média de idade observada foi 61,7±14,5 anos e 33,3% dos pacientes tinha entre 45 e 60 anos (Tabela 3). Nesta etapa o instrumento apresentou uma média de CVC de 0,96, calculado pelas respostas dos pacientes. Sendo assim, ao final do pré-teste, verificou-se boa aceitação e compreensão do instrumento entre os pacientes, independente do grau de escolaridade, não havendo necessidade de modificação dos itens na versão final e, portanto, o novo instrumento (V10) (Figura Central). A síntese das versões de construção, adaptação e validação do EmpoderACO se encontra disponível no Material suplementar A.

**Tabela 3:** – Características da amostra do pré-teste, Belo Horizonte, 2019

Características	Amostra (n=30)
**Sexo, n (%)**	
Feminino	15 (50,0%)
Masculino	15 (50,0%)
**Média de idade em anos (desvio padrão)**	61,7 (±14,5)
**Idade (anos), n (%)**	
< 45	5 (16,7%)
45-60	10 (33,3%)
61-75	7 (23,3%)
≥ 76	8 (26,7%)
**Escolaridade, n (%)**	
Ausência de experiência escolar	2 (6,7%)
Ensino fundamental incompleto	15 (50,0%)
Ensino fundamental completo	7 (23,3%)
Ensino médio	5 (16,7%)
Ensino superior	1 (3,3%)

**Figura Central:**
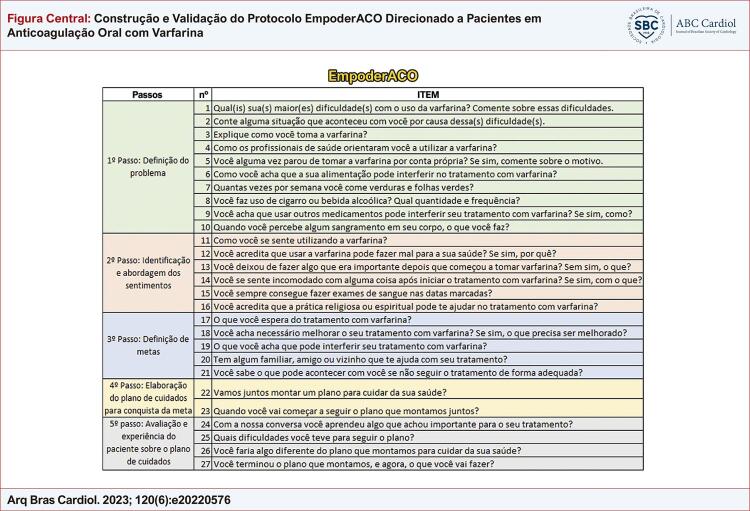
: Construção e Validação do Protocolo EmpoderACO Direcionado a Pacientes em Anticoagulação Oral com Varfarina

Versão final (V10) instrumento EmpoderACO. Fonte: Elaborado para fins deste estudo.

## Discussão

Instrumentos como o EmpoderACO podem ser de grande utilidade no contexto da saúde pública no Brasil, pois até o momento não se identificou instrumento pautado no empoderamento e mudança de comportamento voltado ao paciente com doenças cardiovasculares. O presente estudo permitiu construir o instrumento cujos desempenhos na relevância, adequação, clareza e validação foram considerados bastante satisfatórios. A elaboração do mapa conceitual que precedeu o desenvolvimento do instrumento possibilitou a identificação de domínios de autocuidado na anticoagulação oral que deveriam ser abordados no EmpoderACO. Todos os 30 itens da versão V9 julgados pelo CJ foram analisados pelo CE interno e os itens foram adaptados, excluídos ou invertidos segundo a sequência do instrumento. Conforme a literatura, os itens que apresentam CVC menor que 0,78 não necessariamente devem ser excluídos, embora obrigatoriamente necessitem sofrer modificações, como é o caso dos itens 4 e 16.^25,35^ Por meio da análise do grau de pertinência da versão V9, feita pelo CJ, foi observado que, de forma geral, a maioria dos itens era representada por mais de uma categoria. Contudo, verificou-se que houve consistência e homogeneidade dos resultados da avaliação.

O CVC calculado pelas respostas dos pacientes no pré-teste apresentou resultado muito satisfatório (0,96).^26,29^ Nenhum paciente fez sugestão de modificação ou acréscimo de questões durante a etapa de pré-teste. Nessa etapa, as modificações devem ser consideradas somente se 15% ou mais dos participantes apresentarem dificuldades de compreensão, conforme proposto pelos autores Ciconelli et al.^36^ e Ramada-Rodilla et al.^37^

Promover mudanças de comportamento como ingesta regular de alimentos ricos em vitamina K, monitoramento frequente dos exames de RNI, evitar a automedicação e realizar inspeção do corpo quanto a sinais e sintomas de hemorragia são exemplos relevantes de ações para monitoramento de efetividade e segurança do tratamento em pacientes em uso de varfarina.^38,39^ A construção de instrumentos com obtenção de dados em saúde possibilita organizar as informações de forma clara e objetiva para uma assistência de qualidade e embasar intervenções em saúde.^40^ Estratégias pautadas no empoderamento que se propõem a elaborar plano de cuidados para pacientes com doenças crônicas demonstraram resultados favoráveis para controle glicêmico, autocuidado e empoderamento dos usuários como apontado pelos estudos de Macedo et al.;^41^ Cortez et al.;^25^ Chaves et al.;^24^ Barbosa et al.^9^ No que se refere ao uso do anticoagulante, estas estratégias são necessárias para aumento da efetividade e diminuição dos eventos adversos associados ao uso da varfarina.^10^

O protocolo EmpoderACO poderá ser aplicado na prática clínica como suporte no cuidado ao paciente sendo utilizado pelos profissionais de saúde e pela equipe multiprofissional de modo a fortalecer a qualidade das intervenções e abordagens educacionais. Como perspectivas desse estudo, podemos citar a realização de novas investigações como a validação do PMC em grupos aleatórios de modo a testar o impacto do EmpoderACO nos desfechos da terapia e segurança, frente a um grupo controle, bem como o emprego em futuros estudos na área da anticoagulação. Dessa forma, poderá ser utilizado para discussões adicionais, aprofundamento da percepção do protocolo pelos profissionais de saúde, avaliação dos resultados e dos impactos clínicos, adesão ao tratamento e promoção da segurança do paciente.

A relevância clínica do EmpoderACO deve-se à possibilidade de o instrumento sistematizar a comunicação, nortear abordagens educacionais multidisciplinares em saúde coletiva, estimular a humanização do cuidado, bem como a abordagem individualizada e centrada no paciente. Além disso, espera-se que o empoderamento do usuário para mudanças de comportamento possa fortalecer a relação profissional-paciente e aumento da compreensão da terapia, o que pode favorecer a adesão ao tratamento. Almeja-se que o instrumento possa auxiliar os pacientes de alta complexidade, em uso de anticoagulantes orais, a se tornarem mais capazes de tomar decisões em prol de seu autocuidado e aprimorar a qualidade do processo assistencial, a fim de melhorar os resultados clínicos, bem como a redução dos eventos adversos associados ao uso de anticoagulantes orais.

O instrumento EmpoderACO apresentou como ponto positivo o cumprimento do requisito de concordância geral mínima de 0,80 para construção e validação de novos instrumentos em todos os graus avaliados: relevância (0,92), adequação (0,92) e clareza (0,91).^26,33^ Outro ponto positivo observado foi facilidade de compreensão do instrumento e boa aceitação pela população que não sabe ler e com baixa escolaridade. Atenta-se como limitação do estudo o fato dos itens com necessidade de reformulação não terem sido submetidos novamente à análise de CVC pelo CJ. Desta forma, não foi possível a mensuração do CVC dos itens adaptados. Entretanto, tais itens foram reformulados conforme as sugestões do mesmo CJ e observou-se que a clareza destes itens não foi comprometida, visto que, na etapa pré-teste houve compreensão adequada dos itens da versão V10 pela população-alvo. Observa-se também limitações inerentes aos dados coletados diretamente com os pacientes, tais como desconforto para o paciente ao responder a uma pergunta, bem como viés das informações coletadas.

## Conclusões

O instrumento EmpoderACO se mostrou adequado e de fácil compreensão pelos usuários de varfarina, sobretudo apresentou potencial para uso em pessoas com baixo grau de escolaridade, podendo ser relevante em públicos com escolaridade semelhante. O emprego do protocolo EmpoderACO no campo da anticoagulação permitirá utilizar dos princípios de problematização, autocuidado, empoderamento e cuidado centrado no indivíduo como estratégias para melhorar os resultados terapêuticos da anticoagulação oral.

## Footnotes

* Material suplementar Para informação adicional, por favor, clique aqui.

## References

[1] Ageno W, Gallus AS, Wittkowsky A, Crowther M, Hylek EM, Palareti G (2012). Oral Anticoagulant Therapy: Antithrombotic Therapy and Prevention of Thrombosis, 9th ed: American College of Chest Physicians Evidence-Based Clinical Practice Guidelines. Chest.

[2] Brasil, Ministério da Saúde, Secretaria de Ciência, Tecnologia e Insumos Estratégicos, Departamento de Assistência Farmacêutica e Insumos Estratégicos (2010). Formulário Terapêutico Nacional 2010: Rename 2010.

[3] Malagutte KNDS, Silveira CFDSMPD, Reis FM, Rossi DAA, Hueb JC, Okoshi K (2022). Quality of Oral Anticoagulation in Atrial Fibrillation Patients at a Tertiary Hospital in Brazil. Arq Bras Cardiol.

[4] Ansell J, Hirsh J, Hylek E, Jacobson A, Crowther M, Palareti G (2008). Pharmacology and Management of the Vitamin K Antagonists: American College of Chest Physicians Evidence-Based Clinical Practice Guidelines (8th Edition). Chest.

[5] Lee TW, Lee SH, Kim HH, Kang SJ (2012). Effective Intervention Strategies to Improve Health Outcomes for Cardiovascular Disease Patients with Low Health Literacy Skills: A Systematic Review. Asian Nurs Res.

[6] Lane DA, Barker RV, Lip GY (2015). Best Practice for Atrial Fibrillation Patient Education. Curr Pharm Des.

[7] Clarkesmith DE, Pattison HM, Khaing PH, Lane DA (2017). Educational and Behavioural Interventions for Anticoagulant Therapy in Patients with Atrial Fibrillation. Cochrane Database Syst Rev.

[8] Praxedes MFDS, Mambrini JVM, Reis AMM, Abreu MHNG, Martins MAP (2020). Assessment of Patient Knowledge on Warfarin: An Item Response Theory Approach. J Clin Pharm Ther.

[9] Barbosa HB, Oliveira JAQ, Costa JM, Santos RPM, Miranda LG, Torres HC (2021). Empowerment-Oriented Strategies to Identify Behavior Change in Patients with Chronic Diseases: An Integrative Review of the Literature. Patient Educ Couns.

[10] Costa JMD, Marcolino MS, Torres HC, Resende RE, Souza RP, Barbosa HC (2019). Protocol of a Clinical Trial Study Involving Educational Intervention in Patients Treated with Warfarin. Medicine.

[11] Martins MAP, Costa JM, Mambrini JVM, Ribeiro ALP, Benjamin EJ, Brant LCC (2017). Health Literacy and Warfarin Therapy at Two Anticoagulation Clinics in Brazil. Heart.

[12] Piatkov I, Rochester C, Jones T, Boyages S (2010). Warfarin Toxicity and Individual Variability-Clinical Case. Toxins.

[13] Martins MA, Carlos PP, Ribeiro DD, Nobre VA, César CC, Rocha MO (2011). Warfarin Drug Interactions: A Comparative Evaluation of the Lists Provided by Five Information Sources. Eur J Clin Pharmacol.

[14] Praxedes MFS, Martins MAP, Mourão AOM, Gomes KB, Reis EA, Souza RP (2020). Non-Genetic Factors and Polymorphisms in Genes CYP2C9 and VKORC1: Predictive Algorithms for TTR in Brazilian Patients on Warfarin. Eur J Clin Pharmacol.

[15] Robson J, Dostal I, Mathur R, Sohanpal R, Hull S, Antoniou S (2014). Improving Anticoagulation in Atrial Fibrillation: Observational Study in Three Primary Care Trusts. Br J Gen Pract.

[16] Sevilla-Cazes J, Finkleman BS, Chen J, Brensinger CM, Epstein AE, Streiff MB (2017). Association between Patient-Reported Medication Adherence and Anticoagulation Control. Am J Med.

[17] Anderson RM, Funnell MM (2010). Patient Empowerment: Myths and Misconceptions. Patient Educ Couns.

[18] Mantwill S, Fiordelli M, Ludolph R, Schulz PJ (2015). EMPOWER-Support of Patient Empowerment by an Intelligent Self-Management Pathway for Patients: Study Protocol. BMC Med Inform Decis Mak.

[19] Bandura A (2004). Health Promotion by Social Cognitive Means. Health Educ Behav.

[20] Small N, Bower P, Chew-Graham CA, Whalley D, Protheroe J (2013). Patient Empowerment in Long-Term Conditions: Development and Preliminary Testing of a New Measure. BMC Health Serv Res.

[21] Funnell MM, Anderson RM (2004). Empowerment and Self- Management of Diabetes. Clinical Diabetes.

[22] Anderson RM, Funnel MM (2002). Using the Empowerment Approach to Help Patients Change Behavior. Practical Psychology for Diabetes Clinicians.

[23] Funnell MM, Tang TS, Anderson RM (2007). From DSME to DSMS: Developing Empowerment- Based Diabetes Self-Management Support. Diabetes Spectrum.

[24] Chaves FA, Cecilio SG, Reis IA, Pagano AS, Torres HC (2019). Translation and Cross-Cultural Adaptation of the Behavior Change Protocol for Educational Practices in Diabetes Mellitus. Rev Lat Am Enfermagem.

[25] Cortez DN, Macedo MM, Souza DA, Santos JC, Afonso GS, Reis IA (2017). Evaluating the Effectiveness of an Empowerment Program for Self-Care in Type 2 Diabetes: A Cluster Randomized Trial. BMC Public Health.

[26] Coluci MZ, Alexandre NM, Milani D (2015). Construction of Measurement Instruments in the Area of Health. Cien Saude Colet.

[27] Chenot JF, Hua TD, Abu Abed M, Schneider-Rudt H, Friede T, Schneider S, Vormfelde SV (2014). Safety relevant knowledge of orally anticoagulated patients without self-monitoring: a baseline survey in primary care. BMC Fam Pract.

[28] Brasil, Ministério da Saúde, Secretaria de Atenção à Saúde, Departamento de Atenção Básica (2014). Estratégias para o Cuidado da Pessoa com Doença Crônica.

[29] Pasquali L (1998). Princípios de Elaboração de Escalas Psicológicas. Rev Psiq Clin.

[30] Snyder CF, Watson ME, Jackson JD, Cella D, Halyard MY, Mayo/FDA Patient-Reported Outcomes Consensus Meeting Group (2007). Patient-Reported outcome Instrument Selection: Designing a Measurement Strategy. Value Health.

[31] Keszei AP, Novak M, Streiner DL (2010). Introduction to Health Measurement Scales. J Psychosom Res.

[32] Alexandre NM, Coluci MZ (2011). Content Validity in the Development and Adaptation Processes of Measurement Instruments. Cien Saude Colet.

[33] Grant JS, Davis LL (1997). Selection and Use of Content Experts for Instrument Development. Res Nurs Health.

[34] Polit DF, Beck CT (2006). The Content Validity Index: Are You Sure You Know What’s Being Reported? Critique and Recommendations. Res Nurs Health.

[35] Shi J, Mo X, Sun Z (2012). Content Validity Index in Scale Development.

[36] Ciconelli RM, Ferraz MB, Santos W, Meinão I, Quaresma MR (1999). Tradução para a língua Portuguesa e Validação do Questionário Genérico de Avaliação de Qualidade de Vida SF-36 (Brasil SF 36). Rev Bras Reumatol.

[37] Ramada-Rodilla JM, Serra-Pujadas C, Delclós-Clanchet GL (2013). Cross-Cultural Adaptation and Health Questionnaires Validation: Revision and Methodological Recommendations. Salud Publica Mex.

[38] Chang CH, Wang YW, Yeh Liu PY, Kao Yang YH (2014). A Practical Approach to Minimize the Interaction of Dietary Vitamin K with Warfarin. J Clin Pharm Ther.

[39] Kleibert KRU, Hermann EF, Nunes PL, Schneider A, Stumm EMF, Colet CF (2020). Polimedicação em Usuários de Varfarina Sódica do Sistema Único de Saúde e Variáveis Associadas. Rev Cienc Saude.

[40] Soares LH, Pinelli FGS, Abrão ACFV (2005). Construção de um Instrumento de Coleta de Dados de Enfermagem em Ginecologia. Acta Paul Enferm.

[41] Macedo MML, Cortez DN, Santos JC, Reis IA, Torres HC (2017). Adesão e Empoderamento de Usuários com Diabetes Mellitus para Práticas de Autocuidado: Ensaio Clínico Randomizado. Rev Esc Enferm USP.

