# Supplementary material for: Construção e Validação do Protocolo EmpoderACO Direcionado a Pacientes em Anticoagulação Oral com Varfarina
Source: Arq Bras Cardiol. 2023 Jun 20;120(6):e20220576. [Article in Portuguese] doi: 10.36660/abc.20220576 (PMC10344350; doi:10.36660/abc.20220576)
Supplement: Translation

# Translation: Construction and Validation of a Protocol Targeting Patients on Oral Anticoagulation with Warfarin

**Authors:** Hannah Cardoso Barbosa, Heloisa de Carvalho Torres, João Antônio de Queiroz Oliveira, Rebeca Priscila de Melo Santos, Josiane Moreira da Costa, Leonardo Gonçalves Miranda, Adriana Silvina Pagano, Marcus Fernando da Silva Praxedes, Maria Auxiliadora Parreiras Martins

**Affiliations:** 1 Brazil Universidade Federal de Minas Gerais, Belo Horizonte, MG – Brazil; 2 Brazil Hospital Risoleta Tolentino Neves, Belo Horizonte, MG – Brazil; 3 Brazil Universidade Federal dos Vales do Jequitinhonha e Mucuri, Teófilo Otoni, MG – Brazil; 4 Brazil Universidade Federal do Recôncavo da Bahia (UFRB), Cruz das Almas, BA – Brazil

**Keywords:** Health Behavior, Health Education, Warfarin, Anticoagulants

## Abstract

**Background:**

Warfarin is an oral anticoagulant that is very useful in preventing thromboembolism, though it is considered a drug with a high risk of causing adverse events. Considering the practical challenges in controlling oral anticoagulation, the patients on warfarin could benefit from educational strategies aimed at behavioral changes, active participation in self-care, and adherence to drug therapy.

**Objective:**

The aim was to construct and validate the EmpoderACO protocol for behavioral changes in warfarin patients.

**Methods:**

The methodological steps were: definition of concepts and domains of self-care, identification of objectives, construction and selection of items, assessment of content validity, and pre-test in the target population.

**Results:**

Relevance, adequacy, clarity, and internal reliability of the instrument’s items were assessed by a multidisciplinary judges committee (JC) through the E-surv web platform, obtaining an average agreement of ≥0.91. The understanding of the instrument measured by the target population revealed adequate clarity with a coefficient average of 0.96.

**Conclusion:**

EmpoderACO can aid in qualifying the communication process between medical professionals and patients, as well as in improving adherence to both treatment and clinical outcomes, and can be replicated in healthcare settings.

## Introduction

Warfarin is a coumarin-derivative oral anticoagulant widely used for primary and secondary prevention against thromboembolism.^1^ Even with the advent of direct oral anticoagulants, warfarin is still the main oral anticoagulant provided by the Brazilian Unified Health System (SUS, in Portuguese).^2^ However, inadequate control of this medication may cause adverse events, such as hemorrhage and thromboembolism, which may occur due to exacerbating the anticoagulant effect or therapeutic failure, respectively.^3,4^ The introduction of educational interventions may contribute to reaching the therapeutic results of this medication, improving the patient’s knowledge of oral anticoagulants and consequently improving adherence and satisfaction with the treatment.^5-8^ Desirable changes in behavior, actions aimed at health education, and the empowerment of patients have been seen as important elements for success in anticoagulant treatments.^9-11^

Treatment requires frequent laboratorial monitoring, and reaching the therapeutic target group may be difficult due to the multiple factors which interfere with the treatment, such as dose-response variability, the influence of genetic polymorphism, the presence of comorbidities, high number of interactions with other medications or foods, low level of health education, and concerns by the patients regarding adverse reactions, which can lead to self-interruption of the medication, and those who may require frequent dose adjustments.^1,7,12-14^ The risk of having adverse effects from the treatment increases when the medication is used incorrectly, such as serious hemorrhagic events, like hemorrhagic and/or thrombotic strokes, these events being triggered by the exacerbation of the anticoagulant effect or by therapeutic failure, respectively.^1,7,11,15,16^In such a context, adherence to pharmacotherapy represents a necessary condition to improve the effectiveness and safety of the treatment, avoiding additional challenges in the patient care process.^16^

Empowerment is quite useful in increasing the feeling of control, self-efficiency, coping ability, management of the treatment, and the individual’s ability to reflect on his/her contribution in the process, as well as to achieve a change in behavior regarding the individual’s own health condition.^17-20^ In the context of chronic diseases, the Behavior Change Protocol (BCP) was proposed, which was originally developed by researchers from the University of Michigan for type 2 diabetes mellitus patients ^21-23^ and was later translated and validated for the Brazilian population.^24,25^Currently, there is a scarcity of instruments and directives that guide practices related to empowerment, self-care, and behavioral changes in patients undergoing anticoagulation treatment. Moreover, they are not always conscious and mobilized regarding the importance of this kind of educational approach.^9,25^

Using standardized strategies based on empowerment may guide health professionals towards more active participation of the patients in self-care and adherence to pharmacotherapy by patients taking oral anticoagulants. Creating a protocol for oral treatment with warfarin, based on principles of behavioral changes guided by the BCP, may promote better clinical results and help systematize the communication channels between patients and health professionals. Moreover, it could also increase the patient’s satisfaction with the treatment, improve adherence, reduce adverse effects, and allow the patient/user to recognize the need for changes in behavior. ^7,26,28^ This study aims to construct and validate the EmpoderACO protocol to produce behavioral changes in patients undergoing oral treatment with warfarin.

## Method

This study was developed in stages, following Coluci et al.^26^ methodology, which are: definitions of concepts and domains of self-care in anticoagulation treatment with warfarin; identification of the instrument’s objectives and selection of items according to the instrument’s objective; evaluation of content validity by the Judges Committee (JC); performance of pre-test validation with warfarin patients; and description of the variables and statistical analysis. The stages of the process took place from December 2017 to June 2019.

The structure of the items in the EmpoderACO protocol follows the five steps for behavioral change, according to the BCP^21-25^empowerment study, as follows: Step 1: Definition of the problem; Step 2: Identification and approach to the feelings; Step 3: Definition of the target(s); Step 4: Elaboration of the care plan for reaching the targets; and Step 5: Evaluation and user experience regarding the care plan. This study was approved by the Research Ethics Committee from the Universidade Federal de Minas Gerais (UFMG), Decision no. 2,018,850, CAAE: 65928316.3.0000.5149. After being informed about the study’s objectives and the nature of the data collection, all participants signed the free and informed consent form.

### Definition of concepts, domains of self-care, and objectives of the instrument

According to Pasquali,^29^ the construction of a conceptual structure is the stage which defines the context of the instrument and supports the development of its dimensionality. Therefore, a conceptual map was produced using the *CmapTolls* program, version 6.02 (2017), to identify the domains of self-care on which the instrument should be based. We identified the need to construct specific items for the target public: patients with cardiopathies with highly complex clinical conditions and specificities regarding oral anticoagulant treatment. The stages were conducted from December 2017 to August 2018. According to a study by Snyder et al.^30^ it is crucial that the objectives of the health instrument be pre-defined before its construction and that these objectives be connected to domains and concepts that will be inserted in the instrument.

### Construction of the instrument and item selection

The construction and validation of the instrument were conducted according to methodological stages proposed in the studies by Coluci et al.^26^ and Pasquali.^29^ The instrument was initially constructed by an internal committee of experts (CE) with broad experience in clinical anticoagulation, consisting of three clinical pharmacists, one nurse, and one linguist capacitated in adapting and validating instruments used in the health area. Meetings were conducted to discuss the pertinence and adequation of each item in the context of oral anticoagulation. The internal CE defined self-care domains, measured in the previous stage of the conceptual map, which would be included in the protocol. The internal CE needed to elaborate 12 new items so that the new instrument could address the target population and aimed at self-care and oral anticoagulation. In this stage, eight protocol versions were constructed (V1-V8) before sending it to the external CE (Supplementary Material A).

The V8 version, defined as the test version, was submitted to the pilot evaluation of the external CE by the *Survey E-surv web platform*. In the pilot evaluation, five health professionals were invited, all with experience and knowledge in anticoagulation, including one physician, two pharmacists, and two nurses. The external CE analyzed each protocol item and suggested new adaptations to the structure and content. After the adaptations had been suggested and deemed relevant by the internal CE, an initial version of the instrument was constructed (V9), which, later was delivered to the JC. These stages were conducted from September 2018 to March 2019.

### Evaluation of the instrument by the JC

The JC consisted of 34 professionals with a multidisciplinary profile, and its function was to judge and analyze all of the protocol’s items. After, the structure and organization of the instrument were tested according to the hypothesis that the chosen items properly represented the domains of the desired construct.^26,29,31^ The JC conducted the analysis by evaluating the content and following the recommendations in the literature regarding the minimum number of judges and participation of experts in the area of measuring instruments.^32^ The analyses by the judges involved qualitative and quantitative procedures.^26^ Professionals were chosen to be part of the JC according to the following criteria: have a degree in the field of health and have knowledge and/or experience with clinical practice in caring for patients with the oral anticoagulant warfarin and/or professionals with experience in the process of adaptation and validation of instruments.

The JC members completed an introductory questionnaire using the same online platform (*Survey E-surv)*. Next, the participants were invited to evaluate the V9 version and register their opinions to evaluate the level of relevance, adequation, and clarity of the instrument. The participants had one month to turn in the evaluations. The JC was also asked to analyze the level of the pertinence of the protocol items and to inform which categories the items could measure. In this analysis of pertinence, the judges had access to the meaning of each category and could select more than one category corresponding to the same item. This analysis aimed to group the items according to the domains of self-care for patients taking oral anticoagulants. Those stages were conducted between March and April 2019.

### Pre-test validation in patients

The semantic analysis and the instrument’s validation were conducted through a pre-test and a field test with the target population using the pre-final version of the instrument (V10). The participants evaluated the clarity of each instrument item, aimed at estimating how understandable the instrument was. This stage was conducted at the anticoagulation clinic of the UFMG Hospital das Clínicas by two researchers from the health area with experience in the application of questionnaires for patients. The pre-test was applied to 30 patients. The researchers read the questions to the participants since some were not literate enough to read. The participants were asked to answer about the clarity of the items according to a Likert scale of three points: a) Very Clear, b) Clear, and c) Not Clear. Those stages were conducted in April and May 2019. After the pre-test, there was no need for modifications in the adapted and constructed items; therefore, the V10 version became the final version of the EmpoderACO protocol.

### Description of variables

The descriptive data from the JC members was collected by applying an initial questionnaire using the *Survey E-surv* web platform. The data included: name, place of work, education, and professional practice experience with warfarin. The evaluations of the instrument provided by the JC were exported from the online platform to a digital spreadsheet using Microsoft Excel (version 2019) for subsequent statistical analysis. All of the data was properly codified to ensure the participants’ anonymity.

During the pre-test, a questionnaire to collect sociodemographic data from the patients was applied, covering sex, age, and education to characterize the sample. This stage took place in June 2019.

### Statistical analysis

The instrument’s validation was computed using the Content Validity Coefficient (CVC), a measure capable of evaluating the relevance and representativeness of the items. A minimum CVC agreement was established, equal to 0.80^33^ and, preferably, higher than 0.90.^34^ To evaluate the relevance of each item of the instrument, the JC judged the items according to the answers: 1=Not relevant, 2=Relevant, 3=Very relevant. Adequation and clarity were evaluated according to a three-point Likert scale: 1=Not adequate, 2=Adequate, and 3=Very adequate (for the level of adequation), and 1=Unclear, 2=Clear, and 3=Very clear (for the level of clarity). The calculation was made by the sum of answers “2” and “3” from each judge for each item in the protocol, and that sum was divided by the total number of judges (adapted from COLUCI et al.^26^). The pre-test stage was conducted with 30 patients, a sampling size which was considered sufficient for such an evaluation.^29^ The same calculation for CVC was used in the pre-test stage applied in the field to evaluate the clarity of the items. The sociodemographic data were tabulated and presented descriptively using absolute and relative frequencies to calculate proportions and measures for a central tendency. This stage took place in June 2019.

## Results

The domains of self-care represented in the conceptual map (Figure 1) were divided by the internal CE into three categories: 1) understanding and satisfaction with the treatment, 2) decrease in adverse effects, and 3) promotion of well-being and health habits. After analyzing the domain and its categories, we realized the need to construct 12 new items for the protocol: 4-10; 13-17, according to Table 1.

**Figure 1:** – Domains of self-care in oral anticoagulant treatment. Source: Created with the use of CmapTolls.

**Table 1:** – Content validity coefficient of the answers from the judges’ committee, version V8

Original items	Level of Relevance (CVC)	Level of adequacy (CVC)	Degree of Clarity (CVC)
1 – What is your greatest difficulty in controlling the anticoagulant?	1.00	1.00	0.94
2 – Can you explain that difficulty?	1.00	1.00	0.94
3 – Tell me about some situation that you went through because of that difficulty.	0.97	0.97	1.00
4 – Do you take warfarin as advised?*	0.91	0.88	0.67**
5 – Have you ever interrupted your warfarin treatment? Why?*	0.91	0.94	0.88
6 – Do you think your eating habits may compromise the treatment with warfarin?*	0.97	0.97	1.00
7 – How often and how many vegetables and green leaves do you eat?*	0.94	0.88	0.97
8 – Do you smoke cigarettes or consume alcoholic beverages? How much and how often?*	0.91	0.82	0.94
9 – Do you believe that other medications may interfere in anticoagulation control?*	0.97	0.97	0.91
10 – What do you do when you notice bleeding?*	1.00	1.00	0.97
11 – How do you feel about having to take anticoagulants?	0.97	0.97	0.94
12 – Do you think that warfarin might cause you harm?*	0.85	0.91	0.97
13 – Did you have to stop doing things you enjoy after you began to take warfarin?	0.85	0.85	0.91
14 – What bothered you the most since you began the treatment with warfarin?	0.88	0.85	0.88
15 – How do you feel about having to do frequent blood collections?*	0.97	0.94	1.00
16 – Do you believe that meditation or praying may improve your treatment?*	0.76**	0.70**	0.82
17 – What are your objectives for having treatment with warfarin?*	0.94	0.91	0.90
18 – What do you think you can do in order to improve your treatment?	0.97	1.00	0.97
19 – What would you change in your life in order to feel better?	0.70**	0.64**	0.67**
20 – What problems might interfere with the treatment and achieving your goals?	0.91	0.94	0.91
21 – Is there anyone who could help with your treatment?	0.91	0.91	0.91
22 – Do you know what might happen if you do not care for yourself?	0.97	0.85	0.73**
23 – Shall we work together to plan to care for your health?	0.94	0.97	0.88
24 – Talk about what you can do, step by step, to improve your treatment.	0.76**	0.79	0.88
25 – What are you really going to do to get better?	0.88	0.88	0.88
26 – When are you going to start?	0.85	0.88	0.88
27 – What have you learned from this experience?	0.91	0.94	0.82
28 – What difficulties did you have in following the plan?	0.91	0.91	0.85
29 – What would you do differently?	0.82	0.85	0.82
30 – Now that you have finished the plan, what will you do?	0.79	0.79	0.82
**CVC average**	**0.92**	**0.91**	**0.91**

CVC: content validity coefficient. * Items totally constructed. ** Items with CVC ≤0.78

Of the 80 professionals invited to participate in the JC, 34 (42.5%) sent evaluations of the protocol, and the number of judges proved adequate, according to recommendations in the literature.^35^ The distribution of participants in professional categories was: 40 (50.0%) pharmacists; 17 (21.3%) nurses; 16 (20.0%) physicians; three (3.7%) nutritionists; three (3.7%) linguists, and one pedagogue (1.3%). Pharmacists were the predominant professional category in the JC, followed by nurses, physicians, nutritionists, linguists, and one pedagogue. The general average of the items evaluated by the JC presented CVC above or equal to 0.91 for all analyses: level of relevance, adequation, and clarity. The exceptions were items 4, 16, 19, 22, and 24, as presented in Table 1. Three items from version V9 were excluded by the internal JC after quantitative and qualitative analysis – items 19, 24, and 25 – for not presenting new contributions to the instrument.

We verified that there was consistency and homogeneity in the analysis of the level of the pertinence of version V9 conducted by the JC. The results of the pertinence are presented in Table 2.

**Table 2:** – Representation of item pertinence by category

Category	Items
Adherence to the anticoagulant therapy’s dose scheme	1-5; 11-13; 15-21; 23-30
Blood collection for the oral anticoagulant monitoring exam (INR)	14
Consumption of foods that interfere with the anticoagulant	1-3; 6-7
Concerns about the occurrence of adverse effects caused by the treatment	3; 5; 8; 10; 12; 15; 22
Drug interaction with warfarin	9
Effectiveness of pharmacotherapy	17-18; 20; 22-26; 29-30
“Others” (Understanding and satisfaction with treatment)	19

INR: International Normalized Relation.

The pre-test stage was conducted with a group of 30 patients, representing a heterogeneous sample in terms of age, sex, and level of education, in which 50.0% of the participants were female, and 50.0% had incomplete Elementary School education. The average age was 61.7±14.5 years, and 33.3% of the patients were between 45 and 60 (Table 3). In this stage, the instrument presented a CVC average of 0.96, calculated based on the patients’ answers. Therefore, at the end of the pre-test, a good level of acceptance and understanding of the instrument was observed among the patients, regardless of the level of education, and there was no need to modify items in the final version (the new instrument - V10) (Central Illustration). The synthesis of the two versions of construction, adaptation, and validation of the EmpoderACO is available in

**Table 3:** – Characteristics of the pre-test sample, Belo Horizonte, 2019

Characteristics	Sample (no. = 30)
**Sex, no. (%)**	
Female	15 (50.0%)
Male	15 (50.0%)
**Average age in years (standard deviation)**	61.7 (±14.5)
**Age (years), n (%)**	
< 45	5 (16.7%)
45-60	10 (33.3%)
61-75	7 (23.3%)
≥ 76	8 (26.7%)
**Education, n (%)**	
No education	2 (6.7%)
Incomplete Elementary School	15 (50.0%)
Complete Elementary School	7 (23.3%)
High School	5 (16.7%)
College	1 (3.3%)

**Central Illustration:** : Construction and Validation of a Protocol Targeting Patients on Oral Anticoagulation with Warfarin

Final version (V10) - EmpoderACO instrument. Source: Produced for this study.

## Discussion

Instruments, such as EmpoderACO, may be very useful in the context of public health in Brazil since, to date, no instruments have been based on empowerment and changes in behavior aimed at patients with cardiovascular diseases. The present study allowed us to build an instrument in which adequacy, clarity, and validation were considered highly satisfactory. The elaboration of the conceptual map that preceded the instrument’s development enabled the identification of the self-care domains of anticoagulation treatment, which EmpoderACO should cover. All of the 30 items of version V9 were analyzed by the internal CE, and the items were adapted, excluded, or inverted according to the sequence of the instrument. According to the literature, the items which presented a CVC of lower than 0.78 did not necessarily need to be excluded, although they did have to be modified, as in the case of items 4 and 16.^25,35^ After the agreement analysis of version V9, conducted by the JC, we observed that, in general, most of the items were represented by more than one category. However, consistency and homogeneity could be verified in the evaluation results.

The CVC calculated by the patient’s answers in the pre-test showed highly satisfactory results (0.96).^26,29^ None of the patients suggested modifications or adding questions during the pre-test stage. In that stage, the modifications must be considered only when 15% or more of the participants had difficulty in understanding and comprehension, according to that defined by Ciconelli et al.^36^ and Ramada-Rodilla et al.^37^

The promotion of behavior changes, such as the regular consumption of foods rich in Vitamin K, frequent monitoring of INR, avoiding self-medication, and conducting the self-body inspection for signs of hemorrhage, are relevant examples of actions necessary to monitor the effectiveness and safety of the treatment of patients with warfarin.^38,39^ The construction of instruments to collect health data allows one to organize the information clearly and objectively, thus contributing to quality care and supporting health interventions.^40^ Strategies based on empowerment which propose the elaboration of a health care plan for patients with chronic diseases, have demonstrated favorable results in terms of glycemic control, self-care, and the empowerment of the users, as discussed in the studies by Macedo et al.;^41^ Cortez et al.;^25^ Chaves et al.;^24^ and Cardoso Barbosa et al.^9^ As far as the use of anticoagulant is concerned, such strategies are necessary to increase effectiveness and reduce adverse effects associated with warfarin.^10^

The EmpoderACO can be used in clinical practice to support patient care and employed by health professionals and multidisciplinary teams to improve the quality of interventions and educational initiatives. The strengths of this study include the performance of new investigations, such as the validation of the BCP in random groups in order to test the impact of EmpoderACO in the outcomes of therapy and safety, as compared to a control group, as well as its use in future studies regarding anticoagulation. Therefore, this study can be used for additional discussions, further examining health professionals’ perceptions of the protocol, evaluating results and clinical impacts, adherence to treatment, and patient safety.

The clinical relevance of EmpoderAco stems from the instrument’s ability to systematize communication, guide multidisciplinary educational approaches in public health, encourage more humanized care, and follow a more individualized approach focused on the patient. Moreover, we hope that patient empowerment may strengthen the doctor-patient relationship and improve one’s understanding of the therapy, thereby increasing adherence to the treatment. We hope that the instrument may help high-complexity patients who take oral anticoagulants to become more capable of making their own decisions in favor of self-care and improve the quality of the care process, thus improving both clinical results and the reduction of adverse events associated with oral anticoagulants.

One positive aspect of the EmpoderACO instrument was compliance with the requirement of a general minimum agreement of 0.80 for the construction and validation of new instruments in all of the evaluated categories: relevance (0.92), adequation (0.92), and clarity (0.91).^26,33^ An additional positive aspect was the easy understanding of the instrument and its satisfactory acceptance by illiterate patients and those with a low-level education. One limitation of the study is that the items that need reformulating were not re-submitted to the CVC analysis by the JC. Therefore, we were unable to measure the CVC of the adapted items. However, these items were reformulated according to the suggestions from the JC, and it was noticed that the clarity of the items was not compromised, given that, in the pre-test stage, the target population showed an adequate understanding of the items in the V10 version. We also observed limitations inherent to the data collected directly from patients, such as the patient’s embarrassment in answering some of the questions and information bias within the collected data.

## Conclusions

EmpoderACO proved adequate and easily understandable by warfarin users and showed potential for use among people with a low-level education. Hence, the use of EmpoderACO proved to be relevant for that population. The use of the EmpoderACO protocol in the field of anticoagulation enables the use of the principles of problematization, self-care, and person-centered care as strategies to improve the therapeutic results of oral anticoagulation.

## Footnotes

* Supplemental Materials For additional information, please click here.
